# Research Progress of the Role of Anthocyanins on Bone Regeneration

**DOI:** 10.3389/fphar.2021.773660

**Published:** 2021-10-29

**Authors:** Wei Mao, Guowei Huang, Huan Chen, Liangliang Xu, Shengnan Qin, Aiguo Li

**Affiliations:** ^1^ Department of Orthopedics, Guangzhou Institute of Traumatic Surgery, Guangzhou Red Cross Hospital, Medical College, Jinan University, Guangzhou, China; ^2^ Department of Clinical Medicine, Guizhou Medical University, Guiyang, China; ^3^ Lingnan Medical Research Center, The First Affiliated Hospital of Guangzhou University of Chinese Medicine, Guangzhou University of Chinese Medicine, Guangzhou, China

**Keywords:** anthocyanins, fracture, bone regeneration, osteogenesis, osteoclastogenesis

## Abstract

Bone regeneration in osteoporosis and fragility fractures which are highly associated with age remains a great challenge in the orthopedic field, even though the bone is subjected to a continuous process of remodeling which persists throughout lifelong. Regulation of osteoblast and osteoclast differentiation is recognized as effective therapeutic targets to accelerate bone regeneration in osteopenic conditions. Anthocyanins (ACNs), a class of naturally occurring compounds obtained from colored plants, have received increasing attention recently because of their well-documented biological effects, such as antioxidant, anti-inflammation, and anti-apoptosis in chronic diseases, like osteoporosis. Here, we summarized the detailed research progress on ACNs on bone regeneration and their molecular mechanisms on promoting osteoblast differentiation as well as inhibiting osteoclast formation and differentiation to explore their promising therapeutic application in repressing bone loss and helping fragility fracture healing. Better understanding the role and mechanisms of ACNs on bone regeneration is helpful for the prevention or treatment of osteoporosis and also for the exploration of new bone regenerative medicine.

## Introduction

Osteoporosis is a condition that bones become weak and brittle, so brittle that a fall or a cough can cause a fracture which is often called fragility fracture. Fragility fractures commonly occur in the hip, wrist, or spine, and hip fracture is associated with significant mortality and morbidity. Osteoporosis is closely related to aging. According to the first report on the prevalence of osteoporosis in China issued by the National Health Commission in 2018, 32% of people aged over 65 suffer from osteoporosis. More seriously, 46.4% of individuals aged over 50 were in the condition of low bone mass, suggesting they were at a high risk of becoming osteoporosis (Wang et al., 2021).

The process of bone maintenance is regulated by bone-forming osteoblasts and bone-resorbing osteoclasts. Bone is highlighted by its unique ability to regenerate throughout adulthood, restoring to a fully functional, pre-injury state (Salhotra et al., 2020). For osteoporosis and fragility fractures, the dysregulation of bone biology in the setting of bone repair is “lack of bone”. The excessive bone resorption guided by osteoclasts and/or the impaired capability of bone formation regulated by osteoblasts attributes the bone loss.

Recently, an emphasis has been placed on the relationship between diet and disease, and pieces of evidence have also emerged from clinical trials demonstrating that a dietary pattern, rich in anthocyanins (ACNs), is related to the reduced risk for chronic diseases, such as cancers, obesity, diabetes, and cardiovascular disease (Khoo and Azlan, 2017; D’cunha et al., 2018). ACNs are a class of naturally occurring compounds and show their positive influences on health owing to their antioxidant, anti-inflammatory, and anti-apoptotic potential in various chronic diseases, especially age-related diseases (Aqil et al., 2012; Huang et al., 2018; Speer et al., 2020). Increasing evidence from experimental, and clinical studies showed the consumption of ACNs rich foods played a role in protecting bone loss and helping the healing of fractures (Hardcastle et al., 2011; Mcnaughton et al., 2011).

## Clinical Therapies of Promoting Bone Regeneration

Clinical strategies used in promoting bone regeneration mainly include bone transplantation, stem cell therapy, physical adjuvant therapy, and local injection of growth factors. These approaches and their current advantages and disadvantages are summarized in Table1.

**TABLE1:** Therapies to promote bone regeneration.

Treatment strategies	Treatments	Treatment principle	Advantages	Disadvantages
Bone transplantation	Autogenous bone transplantation Fillingham and Jacobs (2016)	The transfer of cancellous or cortical bone from one part of the body to another	1. Abundant living cells	1. Less available bone source
			2. Low Immunogenicity	2. Poor osteogenesis of donor tissue leads to failure
			3. Low risk of virus transmission	—
			4. Success rate of 80–90%	—
	Allograft bone transplantation Gómez-Barrena et al. (2015)	Obtained from another person	Suitable substitute for autogenous bone	Prolonged operation time and pain
Stem cell therapy	Bone mesenchymal stem cells (BMSCs) Casati et al. (2019)	Interact with a variety of growth factors to promote differentiation of osteoblasts	Wide application	1. Low accessibility
				2. Lack of standardized isolation
				3. Poor long-term stability
	HUC-MSCs (L et al., 2019)	Indirectly promotes bone formation by promoting angiogenesis	1. Wide sources	Low application in bone regeneration
			2. Low risk of infection	
			3. Less immunogenicity	
	PCs Casati et al. (2019)	Periosteum-derived cells (PDCs) were implanted into the defect using scaffolds	1. Strong bone regeneration ability	1. Bionics research is still in its infancy
			2. Be widely used in the treatment of bone nonunion	2. Material selection need to be improved
Drug treatments	rhBMP Nishimura et al. (2008)	1. Promote the differentiation of MSCs into osteogenic and chondrogenic lineages	3. Promote bone regeneration and accelerate healing	1. Expensive treatment
		2. Promote chondrocyte hypertrophy differentiation		2. The spread of rhBMP can lead to ectopic bone formation
		3. Promote callus remodeling		3. Natural bone resorption
		—		4. Soft tissue swelling
		—		5. Dissolve the bone
	PTH James et al. (2016)	1. Promote proliferation	1. Reduce the risk of fractures	High dose injection of PTH induce catabolic reaction leads to fracture healing and repairing
		2. Delay chondrocyte hypertrophy	2. Promote callus formation	
		—	3. Reduce healing time	
Physical adjuvant therapy Padilla et al., (2016), Bhavsar et al., (2020)	Low intensity pulsed ultrasound	Accelerate the repair of fracture injury through external stimulation	Promote angiogenesis and remodeling in callus	Poor healing results in many cases

Autogenous, allograft, and bone grafted substitutes are widely used in the treatment of posttraumatic conditions such as fracture, delayed union, and nonunion (Baldwin et al., 2019). Available in abundant living cells and various growth factors that facilitate the osteogenic differentiation of stem/progenitor cells, the autogenous bone graft is regarded as the gold standard (Gómez-Barrena et al., 2015; Baldwin et al., 2019). Nevertheless, problems like the limited quantity of bone available for harvest make autograft a less-than-ideal option for individuals with osteoporosis (Fillingham and Jacobs, 2016). Allograft and bone graft substitutes provide viable alternatives due to their convenience, abundance, and lack of procurement-related patient morbidity (Baldwin et al., 2019).

Stem cells, including bone mesenchymal stem cells (BMSCs), Human umbilical cord mesenchymal stem cells (HUC-MSCs), and periosteal cells (PCs), owing to their multipotency, anti-inflammatory, and immune-modulatory properties, have been applied in bone repair. BMSCs are the most commonly used stem cells in the field of bone regeneration (L et al., 2019). Endogenous BMSC activation or exogenous BMSCs are utilized for the repair of long bone and vertebrae fractures due to osteoporosis or trauma (Gómez-Barrena et al., 2019). HUC-MSCs Indirectly increased bone formation by promoting angiogenesis, but it is relatively rare for their use on bone regeneration (Li et al., 2016). PCs were widely used in the treatment of bone nonunion and especially used as a source of cells for tissue engineering of bone or cartilage (Li et al., 2016; Duchamp De Lageneste et al., 2018). For stem cell therapy, scaffolds or biomaterials are normally needed to improve their efficacy and stability.

Recombinant human bone morphogenetic protein (rhBMP) is the only osteoinductive growth factor as a bone graft substitute applied in the clinical setting (James et al., 2016). BMP-2 could improve the impaired fusion capacity for some patients, thereby decreasing the prevalence of repeated surgical re-entry, trauma, complications, and additional medical cost (Cahill et al., 2011). However, considering that the delivery of rhBMP often exceeds the physiological dose, this not only leads to the high cost of treatment, but also results in the spread of rhBMP that can lead to adverse effects such as heterotopic bone formation, natural bone resorption, soft tissue swelling, and bone lysis (James et al., 2016)**.** Parathyroid hormone (PTH) provides anabolic therapy for osteoporosis clinically as it has been documented to increase bone mineral density and to reduce the rate of fractures in patients with osteoporosis and also improve fragility fracture-healing (Goltzman, 2018). Systemic injections of parathyroid hormone (PTH) also promoted the proliferation of chondrocytes and osteoblasts (Peichl et al., 2011). However, continuous high-dose injection of PTH could induce a catabolic reaction, which is not conducive to fracture healing and repair (Wojda and Donahue, 2018). In addition, consideration must be given to the instability and variability of growth factors after being injected.

Electrical stimulation (EStim) has been proven to promote bone healing in experimental settings and has been used clinically for many years. Low intensity pulsed ultrasound is the most widespread and studied technique which could accelerate fracture repair in some cases. However, it has not become a mainstream clinical treatment due to the great variation in methods reported, and the inconsistent results associated with this treatment approach (Padilla et al., 2016; Bhavsar et al., 2020).

By now, clinical treatment options for bone regeneration are relatively limited, so it is necessary to develop and improve drugs that are more effective, more economic, and have fewer side effects.

## Anthocyanins and Their Biological Effects on Chronic Diseases

ACNs are a class of water-soluble natural pigments that are prominent in colored plants and belong to flavonoid compounds. More than 635 ACNs have been identified based on the number and location of hydroxyl and methoxy groups (Wu and Prior, 2005; Mulabagal and Calderón, 2012; Sehitoglu et al., 2014; Wallace and Giusti, 2015; Smeriglio et al., 2016; Cerletti et al., 2017; Khoo and Azlan, 2017; Li et al., 2017). Different ACNs may exhibit different bioactive chemical structures due to their specificity. They are derived from the flesh, skin, roots of many colored fruits and vegetables. Anthocyanins, particularly glucosides and galactosides of cyanidin, peonidin, delphinidin, petunidin, Pelargonidin, and malvidin are responsible for the final color of the berries (Millar et al., 2017), and the information of these six major ACNs is summarized in Table 2.

**TABLE 2:** The basic information of six major anthocyanins.

Anthocyanin	Formula	CAS number	Sources	Biological effects	Molecular structure
Delphinidin	C₁₅H₁₁ClO₇	528-53-0	Berries and red wine	Antioxidant; Anti-inflammatory	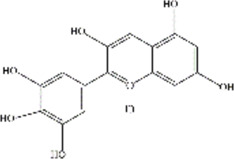
Petunidin	C16H13O7	1,429-30-7	Purple potato and black goji	Antioxidant	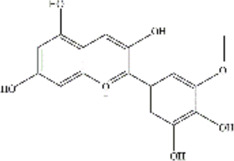
Malvidin	C₁₇H₁₅ClO₇	643-84-5	Blueberries	Apoptosis-inducing; Antioxidant; Anti-tumorogenesis	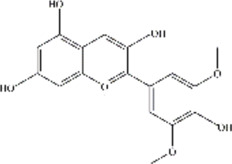
Cyanidin	C15H11O6	13,306-05-3	Cherries	Antioxidant; Anti-angiogenic; Antiviral	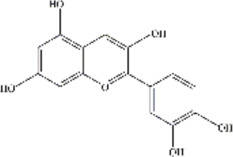
Peonidin	C16H13O6	134-01-0	Berries	Antioxidant; Apoptosis-inducing	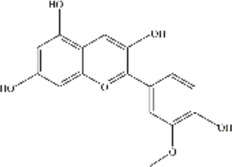
Pelargonidin	C15H11O5	7,690-51-9	Stawberries	Antioxidant	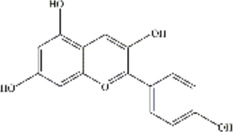

ACNs are well documented for their antioxidant, anti-inflammatory, and anti-apoptotic effects on human health (Henriques et al., 2020; Kozłowska and Dzierżanowski, 2021; Liu et al., 2021; Pérez-Torres et al., 2021; Vega-Galvez et al., 2021). ACNs could be rapidly absorbed in the stomach and detected in the blood and urine (Fang, 2014; Tian et al., 2019), so these pigments are recognized as one of the leading nutraceuticals for prolonging health benefits through the attenuation of chronic, non-communicable diseases, including cancers, obesity, diabetes, cardiovascular diseases, and neurodegenerative diseases.

ACNs have the potential anti-tumor effects for their anti-carcinogenic activities in the initial stage of tumorigenesis, the cancer formation stage, and the cancer development stage (Lin et al., 2017). Delphinidin strongly inhibited cell transformation and migration during tumorigenesis of various cancers (Yun et al., 2009; Ozbay and Nahta, 2011), and it significantly inhibited proliferation and induced apoptosis in osteosarcoma (OS) cell lines (Kang et al., 2018). In addition, delphinidin also showed its promise as a potential chemotherapeutic agent by blocking the development and progression of tumors by inducing apoptotic cell death of osteosarcoma cells (Lee et al., 2018).

ACNs also showed their biological effects on anti-inflammation and anti-oxidative stress (Lee et al., 2017; Samarpita et al., 2020; Tsuda et al., 2000; Choi et al., 2010; Jakesevic et al., 2011; Mane et al., 2011; Kim et al., 2013; Fintini et al., 2020; Casati et al., 2019). With the increase of age, low-grade inflammation and the production of reactive oxygen species (ROS) increased which were involved in the imbalance of bone homeostasis (Domazetovic et al., 2017). Cyanidin could be used for rheumatoid arthritis (RA) treatment as Interleukin 17A/IL-17 receptor A (IL-17/17RA) signaling targeted therapy and could alleviate clinical symptoms, synovial growth, immune cell infiltration, and bone erosion in adjuvant-induced arthritis (AA) rats (Samarpita et al., 2020; Samarpita and Rasool, 2021). Anthocyanins could reduce oxidative stress *in vivo* and *in vitro* (Ali et al., 2018; Ullah et al., 2019; Chen et al., 2020). Delphinidin repressed pathological cardiac hypertrophy by modulating oxidative stress through the AMPK/NADPH oxidase (NOX)/mitogen-activated protein kinase (MAPK) signaling pathway (Chen et al., 2020). The significant increase in the intracellular ROS levels induced by tert-butyl hydroperoxide was prevented by Delphinidin-3-rutinoside treatment (Casati et al., 2019).

However, there are controversial reports of the role of delphinidin on apoptosis. Delphinidin enhanced β2m-/Thy1+ bone marrow-derived hepatocyte stem cells (BDHSCs) survival by inhibiting transforming growth factor-β1 (TGF-β1)-induced apoptosis via PI3K/AKT signaling pathway (Chen et al., 2019), but it induced apoptosis of human osteosarcoma cells with compromising the cellular protective mechanisms (Lee et al., 2018). Therefore, the role of ACNs on apoptosis needs to be explored in the future to better understand the effects of ACNs on apoptosis.

## Role of Anthocyanins on Bone Regeneration

Increasing evidence has demonstrated the beneficial role of ACNs on bone health (Zheng et al., 2016; Melough et al., 2017; Shimizu et al., 2018; Speer et al., 2020). Many ACNs could promote the differentiation of mesenchymal stem cells into osteoblasts and/or inhibit osteoclastogenesis (Park et al., 2015; Dou et al., 2016; Sakaki et al., 2018; Nagaoka et al., 2019a; Nagaoka et al., 2019b; Casati et al., 2019; Saulite et al., 2019; Domazetovic et al., 2020; Hu et al., 2021; Imangali et al., 2021; Karunarathne et al., 2021). In this review, the effects of different ACNs on bone regeneration were described in detail respectively as below and summarized in Table 3.

**TABLE 3:** The roles of anthocyanin in bone regeneration.

Anthocyanin	Functions	*In vitro*	*In vivo*	
—	—	—	Animal model	Micro-CT
Delphinidin	1. Stimulate bone formation	1. BMP2, Runx2, Osx, OCN ↑	OVX rat	1. BV/TV↑
				2. Tb.Th↑
	—	—		3. Tb.N↑
	2. Inhibit bone resorption	2. NF-κB, c-Fos, NFATc1↓		4. Tb.Sp↓
		MMP9, CTSK, DC-stamp↓		5. ES/BS↓
	—	—		6. N.Oc/BS↓
Delphinidin-3-rutinoside	Enhance osteoblast proliferation	CoL1, OCN, ALP↑	—	—
Petunidin	1. Suppress bone resorption	1. >5 μg/ml	Osteopenic mouse model	1. BV/TV↑
		c-Fos, NFATc1↓		2. Tb.Th↑
		MMP9, CTSK, DC-stamp↓		3. Tb.N↑
	—	—		4. Tb.Sp↓
	2. Accelerate osteogenesis	2. >16 μg/ml		5. Oc.S/BS
		BMP2, OCN↑		—
Malvidin	Stimulate bone formation	BMP2, Runx2↑	—	—
			—	—
Cyanidin Chloride	Protect against bone loss	1. NF-κB↓	OVX-induced osteoporosis mouse model	1. BV/TV↑
		2. IκB-α↑		2. OcS/BS↑
		3. ERK↓		3. N.Oc/BS↑
		4. NFATc1, c-Fos↑		—
Cyanidin-3-glucoside	1. Enhance osteoblast proliferation	1. OCN, ALP, Runx2↑	—	—
		—		
	2. Inhibit bone resorption	2. c-Fos, NFATc1↓		
		CTSK, OSCAR, Tm7sf4, Atp6v0d↓		

**FIGURE 1:**
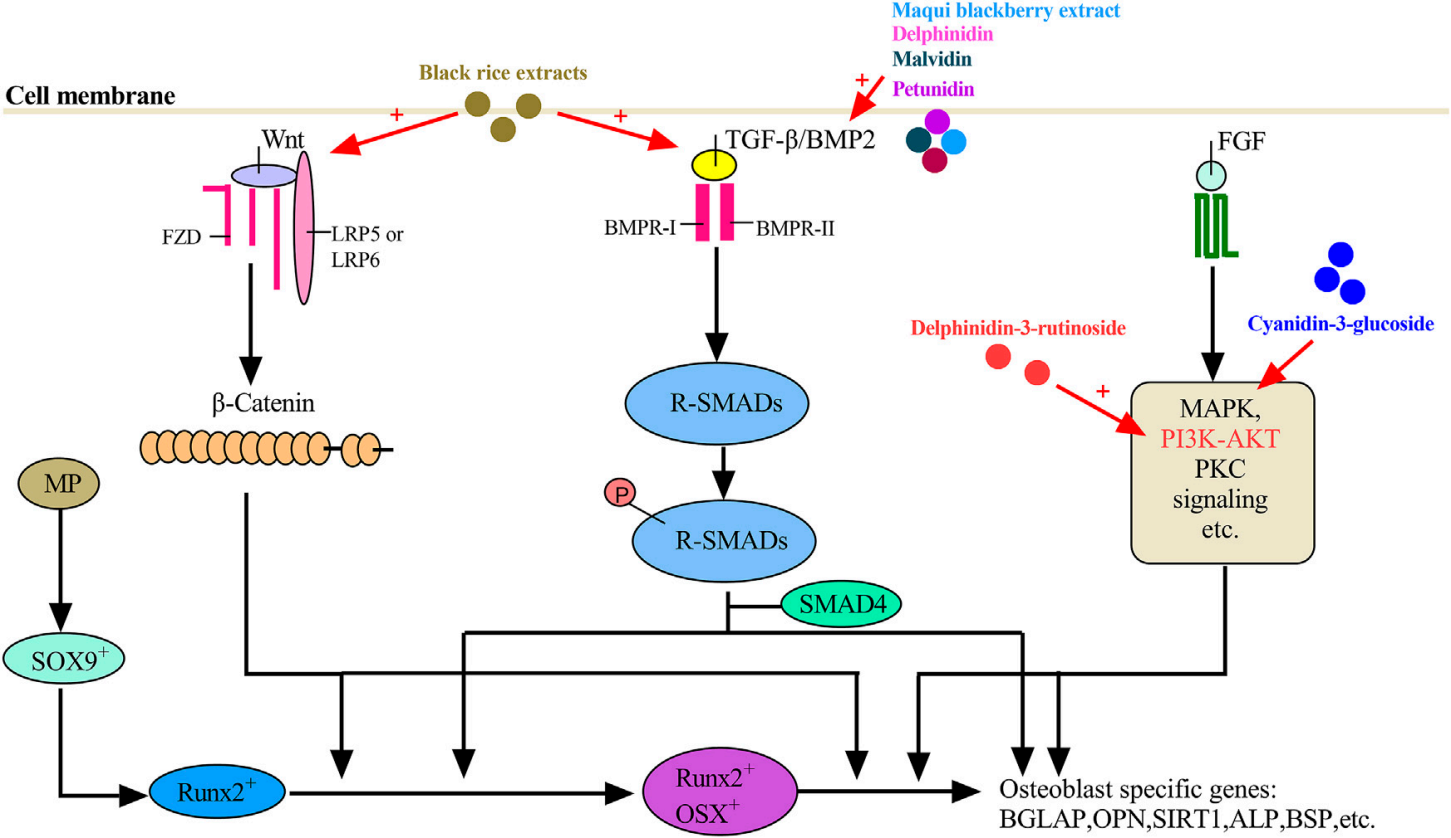
A proposed working model for the promotion of anthocyanins on osteogenesis.

The molecular mechanisms of ACNs underline bone regeneration have also been explored. For osteogenesis, as shown in Figure 1, there are three major pathways involved, including the BMP2 pathway, WNT-β catenin pathway, and FGF pathway. The pathways involved in the differentiation of the osteoblast lineage normally function in a coordinated manner. For example, BMP2 promotes osteoblast differentiation by targeting Runx2 downstream (Salhotra et al., 2020). As shown in Figure 1, the role of Delphinidin-3-rutinoside and Cyanidin-3-glucoside on osteoblast differentiation is mainly by activating the fibroblast growth factor (FGF) pathway, while the mechanisms of other ACNs, including delphinidin, malvidin, and petunidin as well as black rice extracts and maqui blackberry extracts, which also accelerating osteogenesis *in vitro*, are not yet explored. The transcription factors Sox9, Runx2, and Osterix (Osx) are three major components that commit stem/progenitor cells to osteoprogenitor cells, and most of ACNs which could promote osteogenesis upregulated the gene expressions of these transcription factors, at least one of them, as well as osteoblastic markers, such as type 1 collagen (Col1), osteopontin (OPN), osteocalcin (OCN), and alkaline phosphatase (ALP).

**FIGURE 2:**
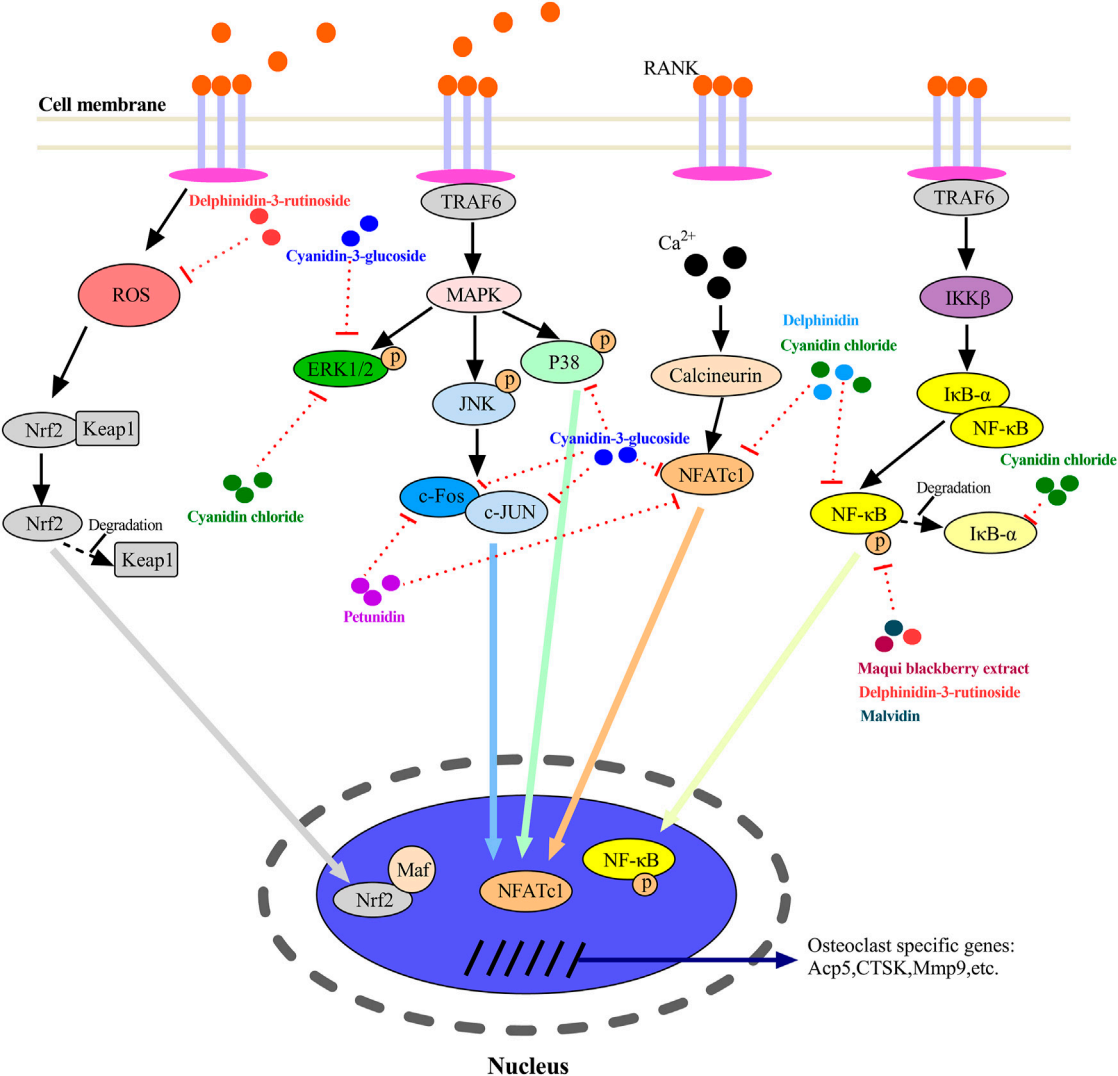
A proposed working model for the inhibition of anthocyanins on osteoclastogenesis.

The mechanisms of most ACNs on osteoclast differentiation were investigated. For osteoclastogenesis, as shown in Figure 2, the c-Fos pathway, NF-κB pathway, JNK pathway, Ca^2+^ pathway, and ROS pathway are four major pathways in osteoclastogenesis. These pathways also interact with each other to be functional. Three subfamilies (P38, ERK1/2, and JNK) of mitogen-activated protein kinases (MAPKs) also play an important role in RANK signal-mediated osteoclast generation (Zhai et al., 2014). The nuclear factor of activated T-cells 1(NFATc1) is a major transcription factor and a key target gene of most of the pathways that regulate osteoclastic differentiation. As shown in Figure 2, the regulation of ACNs which suppressed osteoclast formation and differentiation is through more than one of these pathways. For example, CC could regulate osteoclastogenesis by repressing the expression of ERK1/2, IKBα, and NFATc1, indicating it is involved in several key pathways. Furthermore, most of ACNs downregulated the expression of NFATc1, indicating that they might be involved in several pathways. Most of ACNs that inhibit osteoclastogenesis downregulated the gene expressions of osteoclast specific markers such as Tartrate-resistance acid phosphatase (TRAP), cathepsin K (CTSK), matrix metalloproteinase (MMP9), and dendritic cell-specific transmembrane proteins (DC-stamp). These substances can enhance the bone resorption activity of osteoclasts, decompose bone matrix proteins and inhibit matrix mineralization.

### Delphinidin

Delphinidin [2-(3,4,5-trihydroxyphenyl) chromenylium-3,5,7-triol], a flavonoid compound rich in berries, represents its role in the protection against bone loss by regulating osteoblasts and osteoclasts (Moriwaki et al., 2014; Nagaoka et al., 2019a; Nagaoka et al., 2019b; Imangali et al., 2021). Maqui blackberry extract (MBE), rich in delphinidin, prevented bone loss in osteopenic conditions by not only inhibiting bone resorption by suppressing the NF-κB pathway but also promoting bone formation by enhancing mineralized nodule formation and upregulating osteoblastic genes including BMP-2, Runx2, Osx, and OCN (Nagaoka et al., 2019a) (Figure 1). Osx is another transcript factor and triggers differentiation of immature osteoblasts to mature osteoblasts and eventually into osteocytes (Sinha and Zhou, 2013). Delphinidin could markedly inhibit the osteoclastic differentiation and prevented bone loss in both RANKL-induced osteoporosis model mice and OVX model mice by suppressing the activities of NF-κB, c-Fos, and NFATc1 (Moriwaki et al., 2014).

Delphinidin-3-rutinoside (D3R) is a simpler delphinidin derivative than nasunin (Azuma et al., 2008). D3R protected mouse embryo osteoblast precursor cells (MC3T3-E1) from oxidative damage, and promoted the osteoblastic differentiation of MC3T3-E1 by the PI3K/AKT pathway and increased Col1α1, ALP, and OCN gene expressions after D3R treatment (Figure 1), suggesting the potential utility of dietary D3R supplement to prevent osteoblast dysfunction in age-related osteoporosis (Casati et al., 2019). D3R also exerted their anti-inflammatory effects in LPS-induced osteoclastogenesis partly by inhibiting nuclear translocation of NF-κB (Figure 2), indicating its potential in suppressing bone resorption (Lee et al., 2014).

### Petunidin

Petunidin, a B-ring 5'-O-methylated derivative of delphinidin, also showed its protection against bone loss and multi-faced function on bone cells. Daily oral administration of petunidin (7.5 mg/kg/day) could prevent bone mass loss in RANKL-induced osteopenic mice *in vivo* (Nagaoka et al., 2019b). *In vitro*, petunidin (>5 μg/ml) significantly suppressed osteoclastic differentiation by downregulating c-Fos, NFATc1, MMP9, CTSK, and DC-stamp mRNA expression in pre-osteoclasts (Nagaoka et al., 2019b) (Figure 2). Conversely, petunidin (>16 μg/ml) stimulated mineralized matrix formation and gene expression of BMP2 and OCN, specific osteoblastic markers, of pre-osteoblasts (Nagaoka et al., 2019b) (Figure 1).

### Malvidin

Malvidin is one of the primary plant pigments mainly existing in fruit skins and abundant in blueberries. Blueberries, consist of malvidin (16%), exerted their anti-inflammatory effects in macrophages by inhibiting nuclear translocation of NF-κB (Lee et al., 2014). Reports on the role of malvidin on bone regeneration are rare by now, but it still showed promising application in promoting bone formation. Malvidin induced a significantly higher accumulation of calcium deposits in MSCs comparing to untreated MSCs, as well as upregulated the osteocyte-specific gene BMP-2 and Runx-2 expression and induced BMP-2 secretion (Saulite et al., 2019) (Figure 1).

### Cyanidin

Cyanidin is an anthocyanin widely distributed in cherries. Cyanidin Chloride (CC) and Cyanidin-3-glucoside (C3G) are two cyanidins that are able to regulate bone homeostasis.

Kinds of literatures on the role of CC in regulating osteoclastogenesis are controversial. Cheng et al. (2018) found that CC inhibited osteoclast formation, hydroxyapatite resorption, and RANKL-induced signal pathways *in vitro* and protected against OVX-induced bone loss *in vivo*, indicating its therapeutic potential for osteolytic diseases. CC inhibited RANKL-induced NF-κB activation, suppresses the degradation of IκB-α, and attenuates the phosphorylation of extracellular signal-regulated kinases (ERK). In addition, CC abrogated RANKL-induced calcium oscillations, the activation of nuclear factor of activated T cells calcineurin-dependent 1 (NFATc1), and the expression of c-Fos (Figure 2). However, Dou et al. (2016) suggested that CC had a dual role in the differentiation of osteoclasts. Only a high dosage of cyanidin (>10 µg/ml) suppressed osteoclastogenesis and osteoclast fusion whereas a low dosage (<1 µg/ml) showed an opposite impact.

C3G has also played a role in bone regeneration by regulating osteoblast and osteoclast differentiation. C3G could improve the proliferation of osteoblasts, and upregulate the expression of osteogenic genes, including OCN, ALP, and Runx2 (Park et al., 2015; Kim et al., 2019; Hu et al., 2021). C3G mainly activated the ERK1/2 pathway to regulate the expression of OCN, enhancing the maturation of osteoblasts and promoting bone nodule formation (Cheng et al., 2018) (Figure 1). C3G has also shown promise in inhibiting bone resorption by regulating osteoclastic differentiation. C3G-rich blackberries treatment at the level of 5% (w/w) may modestly reduce OVX-induced bone loss evident by improved tibial, vertebral, and femoral BMD values, and tibial bone microstructural parameters (Kaume et al., 2015). C3G significantly reduced the expression of osteoclastic differentiation markers including CTSK, Osteoclast-associated receptor (OSCAR), transmembrane 7 superfamily member (Tm7sf4) and ATPase, H+ transporting, lysosomal 38kda, V0 subunit d2 (Atp6v0d2), and significantly inhibited the nuclear translocation of c-Fos and NFATc1 (Park et al., 2015) (Figure 2). Furthermore, C3G considerably reduced the induction of extracellular signal-regulated kinase, c-Jun N-terminal kinase, and p38 mitogen-activated kinases activation, which were major pathways regulated by RANKL in osteoclast precursor cells (Figure 2). In the process of osteoclast formation induced by RANKL, NF-κB and ERK/MAPK were activated by RANKL, while C3G attenuated the induction of RANKL in cultured cells *in vitro*, suggesting that C3G could inhibit the generation of osteoclasts (Aqil et al., 2012) (Figure 2).

## Conclusion

In recent years, increasing studies have shown that anthocyanins display their beneficial role on bone formation, including upregulating the osteoblastic genes, promoting the proliferation of osteoblasts and enhancing the mineral nodule formation. Also, they play an important role in inhibiting osteoclastogenesis, able to protect against bone mass loss in osteopenic conditions. Nevertheless, reports of these pigments as therapeutic applications on bone homeostasis, especially on fracture healing, are limited and role of these pigments on bone homeostasis need to be further explored.

## Perspective of Anthocyanins

As a class of natural compounds, ACNs are rich in dietary sources, and their use in the prevention and treatment of adverse health events deserves attention. ACNs are a safe and inexpensive way to prevent diseases with minimal side effects. In the light of the molecular mechanisms, it is possible to find new targets for treating bone-related diseases such as fragility fractures in the future, and provide a new perspective for therapies. Most studies have been conducted *in vitro* or in animal models, while ACNs have rarely been studied in humans. In the future randomized controlled trials are needed to determine the role and mechanism of ACNs in human bone health. Further studies on the preventive dose of ACNs for bone health should standardize the amount of anthocyanin-rich fruits and vegetables consumed by humans. In addition, in order to better apply anthocyanin in orthopedic clinics, it is necessary to further study the role of anthocyanin in bone health, including dose, administration mode, toxicity, and side effects, etc. More importantly, Random Clinical Trial is needed to establish the role and mechanism of anthocyanin.
